# Comparative Evaluation of Electronic Apex Locators and Radiovisiography for Working Length Determination in Primary Teeth *in vivo*

**DOI:** 10.5005/jp-journals-10005-1346

**Published:** 2016-06-15

**Authors:** Ahsan Abdullah, Neerja Singh, Monika S Rathore, Shobha Tandon, Balakrishnan Rajkumar

**Affiliations:** 1 Senior Lecturer, Department of Pedodontics and Preventive Dentistry Post Graduate Institute of Dental Sciences, Lucknow, Uttar Pradesh, India; 2 Professor, Department of Pedodontics and Preventive Dentistry, Babu Banarasi Das College of Dental Sciences, Lucknow, Uttar Pradesh, India; 3 Professor, Department of Pedodontics and Preventive Dentistry, Babu Banarasi Das College of Dental Sciences, Lucknow, Uttar Pradesh, India; 4 Professor, Department of Pedodontics and Preventive Dentistry, Babu Banarasi Das College of Dental Sciences, Lucknow, Uttar Pradesh, India; 5 Professor, Department of Endodontics and Conservative Dentistry, Babu Banarasi Das College of Dental Sciences, Lucknow, Uttar Pradesh, India

**Keywords:** Apex locators, Dentaport ZX, Propex II, Pulpectomy, Radiovisiography, Working length.

## Abstract

**Aims:** The aim of this study was to evaluate the effectiveness of two different varieties of electronic apex locators and radiovisiography (RVG) for working length determination in primary teeth.

**Materials and methods:** A total of 30 primary teeth indicated for pulpectomy in children aged 3 to 8 years were randomly selected and subjected to working length determination using two varieties of electronic apex locators and RVG separately. The data were then subjected to statistical analysis.

**Results:** A very strong correlation between electronic measurement methods and RVG length was observed.

**Conclusion:** Radiovisiography and apex locators are equally effective in determining working length in primary teeth.

**How to cite this article:** Abdullah A, Singh N, Rathore MS, Tandon S, Rajkumar B. Comparative Evaluation of Electronic Apex Locators and Radiovisiography for Working Length Determination in Primary Teeth *in vivo.* Int J Clin Pediatr Dent 2016;9(2):118-123.

## INTRODUCTION

Determining accurate root length is an important part of successful root canal therapy in primary teeth in order to minimize periapical injury and damage to succedaneous tooth bud. In primary teeth, it is important to estimate the exact working length during endodontic therapy to avoid injury to the succedaneous tooth bud. Numerous techniques have been proposed for determination of the same. It can be done clinically by tactile method, radiography, apex locators, paper points, etc.

A technique to be used in working length determination of root canals of primary teeth should give precise and reproducible results.^1^ Being relatively simple, many clinicians still practice tactile perception as an adequate means to detect working length. However, it is generally inaccurate in root canals with constricted canals, excessive curvatures, and root resorption.

Conventional radiographic method described by Ingle has been one of the most popular diagnostic tools for determining working length in endodontics. However, it is only able to provide a two-dimensional (2D) image. The accuracy is difficult to be achieved in this technique, because presence of lateral canals/foramina or an apical constriction may not be identified. Especially in primary teeth where even physiological root resorption is mostly oblique and not horizontal in nature, one cannot rely on a 2D image.

Furthermore, recent technological advances have turned digital radiography into a viable option for the determination of endodontic working length. The reliability of digital radiography is seemingly comparable to or even better than that of conventional radiography.^2^ Other studies, however, reported that conventional radiography is more accurate in comparison to older digital radiographic systems.^3,4^

According to previous studies, conventional radiography yields an 82% precision, whereas in a study done by Olson et al,^5^ electronic measurement is closer to 95%. Comparison between the two techniques shows apex locators to be more accurate and more reliable than radiography for determining working length.^1^ This is primarily due to the fact that electronic measurement is an objective technique, whereas radiography is a subjective technique. This was proven as early as 1983 in the study by Gelfand et al^6^ in which almost 22% of operators disagreed with themselves while examining a set of X-rays for the second time.

These findings clearly indicate that serious consideration should be given to the use of electronic measurement devices as a primary means of determining the root canal length during endodontic procedures. This shift to electronic measurement is also dictated by the difficulty on some teeth in obtaining a radiograph that can be exploitable clinically due to the presence of dental and/or anatomical obstacles blocking or blurring the view of the root apex on the radiograph. However, radiographs remain of paramount importance in endodontic procedures because electronic measurement devices do give accurate estimates of root canal length without doubt, but absolutely no formation on the shape of the root, number of root canals, or direction of curvatures.

Contrary to this, another anatomical factor of grave concern during endodontic procedures in deciduous teeth happens to be natural apical root resorption and destruction of the natural apical constriction. This will in turn create difficulties in locating a biologically acceptable landmark at which to end our treatment. Resorptive processes generally produce uneven root ends, which yield an unclear radiological image with little or no clue to the endpoint of the root canal. Even more, this appearance is only visible in the mesiodistal plane and mostly blurred lingually and buccally.

Thus, the current trial aims to be an attempt to clarify the ongoing debate by comparatively evaluating the effectiveness of measuring working length in primary teeth using radiovisiography (RVG) and two different types of electronic apex locators.

## MATERIALS AND METHODS

The present study was conducted in the Department of Pedodontics and Preventive Dentistry, Babu Banarasi Das College of Dental Sciences, Babu Banarasi Das University, Lucknow, Uttar Pradesh, India, after gaining clearance from the Institutional Ethical Committee and written consent from parents/guardians.

### Sample

For comparison of working length, a total of 30 primary teeth indicated for pulpectomy in a population of 14 children aged 3 to 8 years were randomly selected. Children with irreversible pulpits in primary maxillary and man-dibular anterior teeth with more than two-thirds of the root length remaining were included in the study. Children with primary anterior teeth with more than two-thirds of root resorption, evidence of root fracture, or dilacerated roots were excluded from the study.

### Groups

During pulpectomy, every single anterior tooth was subjected to working length determination by all three modalities for a comparative diagnostic efficiency. Consequently, three groups were established. Group I included working length determination in primary incisors by means of RVG. Group II included working length determination using Propex II and group III included working length determination using Denta-port ZX.

### Methodology

First, the selected primary anterior tooth was isolated, then access cavity was prepared followed by pulp extirpation and irrigation with normal saline. Then each of the selected teeth was subjected to working length determination by all three test modalities. Numerical values were documented and subjected to statistical analysis.

The digital radiographs taken during the course of this study were obtained using a computed dental radiography system and #1 sensor (CDR-Schick Technologies Inc., Long Island City, New York, USA) and X-ray equipment (Gnatus XR 6010; Gnatus, Ribeirao Preto, Sao Paulo, Brazil) installed in our department. The digital images were stored in TIFF format for further analysis.

**Fig. 1:**
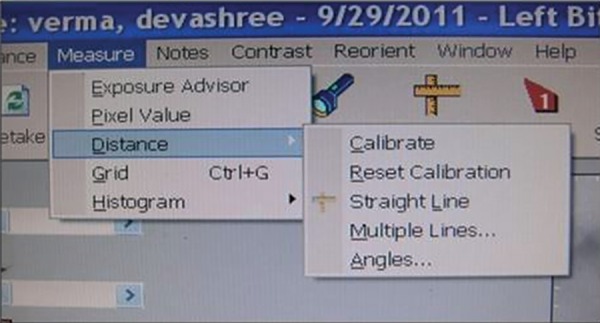
First calibrated step for working length determination in radiovisiography

**Fig. 2:**
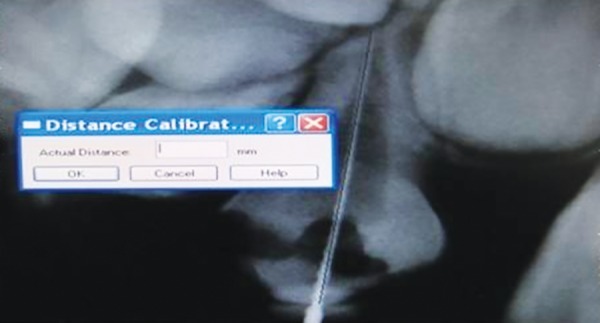
Entering the actual length of file

**Fig. 3:**
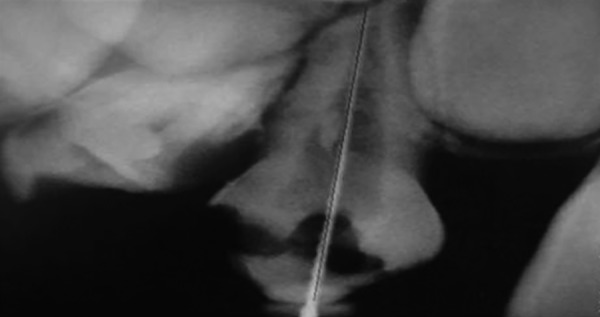
Double click of mouse to get the actual working length

The working length was measured directly on the screen of a high-resolution 17'' monitor with 100% zoom magnification. The measurement method was the electronic ruler of the proprietary CDR system software (version 2.6; Schick Technologies Inc.). Using the left mouse button, a two-click measurement was performed for tooth length determination: One click at the visible edge of the crown and the other at the root apex. Prior to the measurements, the electronic ruler was calibrated by measuring an object of known length, a #20 K file (Mani). Enhancement features, such as brightness and contrast, were not used for the on-screen measurement (Figs 1 to 3).

Subsequently, the same tooth was subjected to working length determination by means of apex locators during the same appointment.

## RESULTS

The working length measured by RVG ranged from 9.90 to 16.00 mm with a mean value of 14.09 ± 1.47 mm (median 14.50 mm). The measurements by Dentaport ZX ranged from 9.00 to 16.50 mm with a mean value of 14.08 ± 1.61 mm (median 14.00 mm), while measurements by Propex II ranged from 9.00 to 16.00 mm with a mean value of 14.05 ± 1.51 mm (median 14.25 mm).

Table 1 shows the range of difference in working length from RVG in two methods being tested. No difference between RVG and Dentaport ZX was observed for nine (30%) cases. A total of 12 (40%) subjects had a difference between 0 and 0.5 mm, whereas a total of nine (30%) cases had a difference of > 0.5 mm. The range of difference between Dentaport ZX and RVG length method was from -2.00 to 1 mm (mean 0.01 ± 0.74 mm).

**Table 1:** **Table 1:** Range of difference in working length from radiovisiography in two methods being tested

*Range of* *difference (mm)*		*Dentaport ZX*				*Propex II*			
		*No.*		*%*		*No.*		*%*	
0		9		30		21		70	
0-0.5		12		40		6		20	
> 0.5		9		30		3		10	

X^2^ = 9.800 (df = 2); p = 0.007

**Graph 1:**
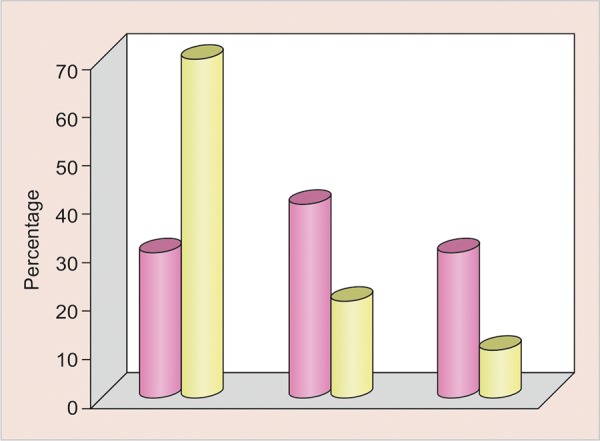
Graphical representation for range of difference from RVG in two methods being tested for working length

The range of difference between RVG length and Propex II was nil for 21 (70%) cases, whereas in 6 (20%) cases, this difference was within 0.5 mm and in 3 (10%) cases the difference was > 0.5 mm. The range of difference between Dentaport ZX and Propex II method was from -1 to 1 mm with a mean of 0.043 ± 0.45 mm.

On comparing the proportion of cases in different range of difference categories between two apex locators, the difference was found to be significant statistically (p = 0.007) (Graph 1).

**Graph 2:**
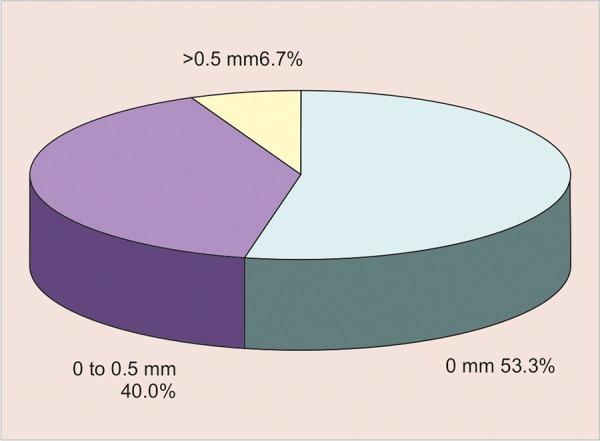
The two Apex locaters showing Range of difference between Dentaport ZX and Propex II methods

Table 2 shows the range of difference between Dentaport ZX and Propex II methods. For majority (53.3%) of comparisons between Dentaport ZX and Propex II methods, the difference was nil, thereby showing a perfect agreement between two techniques for 16 (53.3%) cases. A discrepancy of up to 0.5 mm was observed in 12 (40%) cases, whereas there were 2 (6.7%) cases where the discrepancy was > 0.5 mm (Graph 2).

**Table 2:** **Table 2:** Range of difference between Dentaport ZX and Propex II methods

*Range of difference (mm)*		*No.*		*%*	
0		16		53.3	
0-0.5		12		40.0	
> 0.5		2		6.7	

**Table 3:** **Table 3:** Mean difference between radiovisiography length and length measured by electronic measurement techniques

				*Length*				*Difference from* *RVG*				*Significance of difference* *(paired t-test)*			
*Sl. no.*		*Method*		*Mean*		*SD*		*Mean*		*SD*		*t*		*p*	
1		RVG		14.09		1.47		–		–		–		–	
2		Dentaport ZX		14.08		1.61		-0.01		0.74		0.074		0.94	
3		Propex II		14.05		1.51		-0.04		0.45		0.526		0.60	

SD: Standard deviation; RVG: Radiovisiography

The mean difference between RVG length and length measured by the two electronic measurement techniques is shown in Table 3. Mean RVG was measured as 14.09 ± 1.47 mm, whereas Dentaport ZX and Propex II measured the length as 14.08 ± 1.61 and 14.05 ± 1.51 mm, respectively. On comparing the length measured by Dentaport ZX to the RVG length, a mean difference of -0.01 ± 0.74 mm was observed, whereas the difference between RVG length and Propex II was -0.043 ± 0.452 mm. On comparing the data statistically, both the groups showed no significant difference from the RVG length.

**Graph 3:**
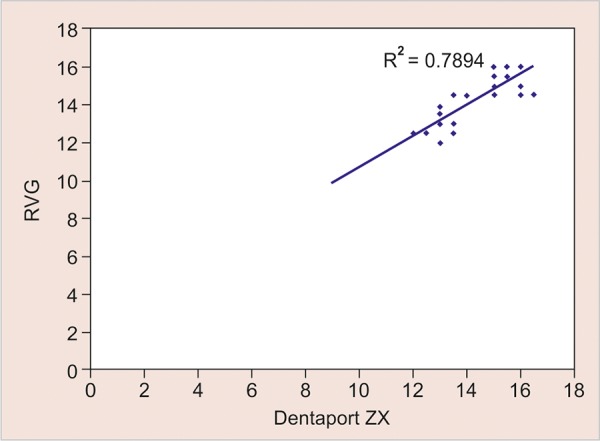
Graphical representation of correlation between the length measured by RVG and Dentaport ZX

**Graph 4:**
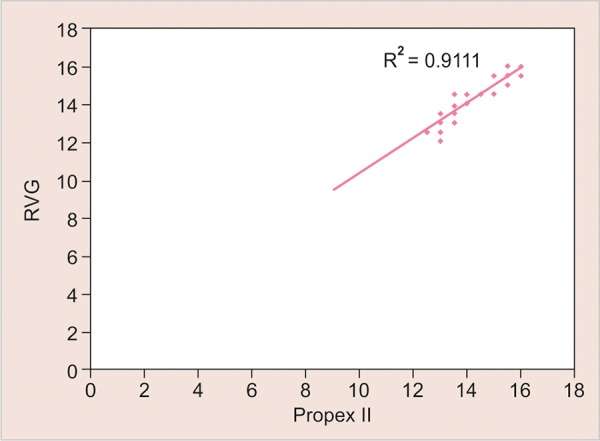
Graphical representation of correlation between the length measured by RVG and Propex II

A very strong correlation between electronic measurement methods and RVG length was observed (r=0.888 and 0.949 respectively), thus showing the possibility of their use as a method to measure the actual length. As we have seen, except for three values (10%) in Propex II and nine (30%) in Dentaport ZX group, all the values had a difference within a range of ± 0.5 mm (Table 2). Given the extent of correlation as observed in Table 4, it is deemed essential if adding a correction factor could reduce the range of difference further. For this purpose, a linear regression was performed (Graphs 3 and 4).

**Table 4:** **Table 4:** Correlation between radiovisiography length and length measured by electronic measurements

		*RVG length*		*Dentaport ZX*		*Propex II*	
RVG length		1		0.888*		0.955*	
Dentaport ZX		–		1		0.949*	
Propex II		–		–		1	

*Is p < 0.001, RVG: Radiovisiography

## DISCUSSION

Working length determination in primary teeth poses a strategic challenge because the physiologic root resorption is not always horizontal but mostly oblique in nature. This poses a serious need to determine the actual extent of the root canal space to be filled in by a resorbable obturating material. This was one of the major reasons to conduct this study using primary teeth.

However, Subramaniam et al^7^ reported an *in vitro* study comparing the tactile sense technique, apex locators, and conventional and digital radiography with the stereomicroscopy to determine the working length in primary single rooted teeth. They did not find statistically significant differences after comparing all the techniques.

The current study, henceforth, was an attempt to statistically evaluate the efficacy of working length determination in primary teeth using RVG and two different generations of apex locators, namely, Dentaport ZX (comprised of two modules: The Root ZX and the Tri Auto ZX), which is a third-generation apex locator, and Propex II, which is a fifth-generation apex locator.

In the methodology, one of the foremost bases to choose digital radiography over conventional radiography was the ability of digital image calibration before each tooth length determination. It was done using the on-screen calibration tool to measure the image of an endodontic file of a known length. It has been shown that calibrated digital measurements are more accurate than uncalibrated measurements.^8^

Kawauchi et al^9^ have demonstrated that image processing by the digital method aids in accurate radio-graphic interpretation by means of calibration. This consequently reduces the margin of error while measuring the working length between the apex and the coronal reference point on the digital tooth image.

However, when Sanabe et al^10^ evaluated *in vitro* the accuracy of primary incisor lengths using RVG by calibration and conventional radiography compared with the actual tooth length, both methods provided similar tooth length measurements which were equivalent to the actual tooth lengths.

During our study, from the adjusted length of tooth radiographically, we subtracted a 0.5 mm “safety factor” to conform to the apical termination of the root canal at the apical constriction. It has been well documented that accepting clinically a 0.5-mm discrepancy between the actual tooth length and lengths estimated on the radiographs, 60% of the measurements obtained with either conventional or RVG images were considered equivalent to the actual tooth lengths.^11^

Radiographs have traditionally been the most common method for determining working length. The reliability of radiographs is compromised because they provide a 2D image of a three-dimensional (3D) object, are technique sensitive, and are subject to observer interpretation. Large tori, dense maxillary bone, or the zygoma can also often superimpose on the image of the root apices, resulting in interpretive error. Since the apical constriction is not visible on radiographs, common practice involves using anatomical averages to estimate this landmark by determining working length to be 0.51 mm short of the anatomical apex. In addition, patients are often apprehensive toward taking radiographs due to radiation concerns.

Due to these shortcomings, electronic methods for root length determination have been developed. Custer et al in 1916 first introduced the concept, which was later revisited by Suzuki et al in 1942 when they observed that a consistent electrical resistance between an instrument in a root canal and an electrode on the oral mucous membrane could be used for measuring canal length.^12,13^

Since that discovery, several generations of electronic apex locators have been developed to improvise on their performance. In our present study, we have used third-and fifth-generation apex locators.

Third-generation devices are largely frequency based and use multiple frequencies to determine the distance from the end of the canal. Certain third-generation devices use a ratio algorithm between two electrical currents and are designed to make accurate readings regardless of fluid electrolytes being present within the canal.

The fifth generation of apex locators can measure pulp space lengths accurately even in the presence of conductive fluids. The device provides the operator with a digital read out, graphic illustration, and an audible signal.

Numerous clinical trials have focused on their ability to prove their efficacy in working length determination in primary and permanent teeth. Goldberg et al^14^ evaluated *in vitro* the accuracy of three electronic apex locators in determining the working length of teeth during retreatment. They found that the ProPex, NovApex, and Root ZX were accurate within 0.5 mm 80, 85, and 95% of the time, and within 1.0 mm 95, 95, and 100% of the time respectively.

During our study, the mean difference between Dentaport and RVG method was 0.01 ± 0.74 mm. The mean difference between RVG and Propex II was 0.043 ± 0.45 mm. This was well in accordance with earlier work conducted by Leonardo et al^15^ who evaluated *ex vivo* the accuracy of two electronic apex locators (Root ZX II-J, Morita Corp. and Mini Apex Locator, Sybron Endo) during root canal length determination in primary incisor and molar teeth with different stages of physiological root resorption. Root canal length was measured both visually (with the placement of 1 mm short of the apical foramen or the apical resorption bevel) and with apex locators. It was found that Root ZX II and Mini Apex Locator proved useful and accurate in locating the apical foramen during root canal length measurement in primary incisors and molars.

In the present study, on using Pearson’s bivariate correlation, a very strong association between electronic measurement methods and RVG length was observed, thus showing the possibility of their use as a method to measure the working length. Except for three values (10%) in Propex and nine (30%) in Dentaport group, all the values had a difference within a range of ± 0.5 mm. It is, therefore, deemed essential if adding a correction factor could reduce the range of difference further. For this purpose, we performed a linear regression. This was done to find out a correction factor that could further reduce the range of difference between electronic methods and RVG length. Both before and after regression, the mean difference from RVG length was higher in Propex method; however, the difference was not significant statistically at either of the two techniques. This shows that the correction factor adds to the efficiency of both the techniques equally.

After linear regression, the difference from RVG length could be reduced in both the techniques. The mean difference between RVG length and Dentaport was observed to be -0.007 ± 0.677 mm, whereas the same between RVG length and Propex was observed to be 0.003 ± 0.440 mm. Thus, both the methods were found to be correlating well with the working length.

This was in accordance with a study conducted by Katz et al^16^ who compared the canal length of primary teeth obtained with the Root ZX and with conventional radiography *in vitro.* They did not find statistically significant differences comparing both methods with the actual length.

In a recent clinical trial conducted by Patino-Marin et al,^17^ it was shown that the most accurate method for determining the working length of the root canals in primary teeth was Root ZX followed by ProPex and the least accurate was conventional radiography.

Being supportive of the above-mentioned evidence, the current investigation also demonstrates that although radiographs have been a hallmark in working length determination, the use of apex locators, especially in pediatric dental patients, may prove indispensible as far as endodontic diagnosis, behavior management of the child, and radiation exposure are concerned.

## CONCLUSION

On the basis of our observation made during the course of study and their analysis, it was seen that apex locators are an effective tool in determination of working length in primary teeth. Two different varieties of apex locators used in the present study were equally effective in doing the same. Moreover, they were as effective as RVG for working length estimation in primary teeth.
